# Machine Learning for Risk Stratification in Acute Pancreatitis: Current Evidence and Future Perspectives

**DOI:** 10.3390/jcm15155967

**Published:** 2026-07-30

**Authors:** Martyna Szczerbakow, Adam Filip Płoński, Piotr Górski, Agnieszka Świdnicka-Siergiejko, Jarosław Daniluk

**Affiliations:** Department of Gastroenterology and Internal Medicine, Medical University of Bialystok, ul. M. Skłodowskiej-Curie 24A, 15-276 Białystok, Poland; adam.plonski@sd.umb.edu.pl (A.F.P.); piotr.gorski@sd.umb.edu.pl (P.G.); agnieszka.swidnicka-siergiejko@umb.edu.pl (A.Ś.-S.); jaroslaw.daniluk@umb.edu.pl (J.D.)

**Keywords:** acute pancreatitis, severity, artificial intelligence, machine learning models, predictors, prognosis

## Abstract

Acute pancreatitis (AP) is one of the most common gastrointestinal diseases and is characterized by a highly variable clinical course ranging from mild self-limiting inflammation to severe disease associated with persistent organ failure and high mortality. Early risk stratification is essential for timely therapeutic decision-making and improved patient outcomes. Although conventional prognostic scoring systems remain widely used, their predictive performance is limited by moderate accuracy, delayed applicability and the requirement for numerous clinical or imaging parameters. Recent advances in artificial intelligence (AI) and machine learning (ML) have enabled the development of predictive models capable of integrating demographic, clinical, laboratory and imaging data to improve early outcome prediction. This review summarizes current evidence on the application of AI and ML in AP, focusing on the prediction of disease severity, organ failure, intensive care unit admission and mortality. We also discuss explainable AI approaches and the methodological strengths and limitations of existing models, including validation, calibration, overfitting and reproducibility. Finally, we highlight future perspectives, including multimodal AI, prospective clinical validation and regulatory considerations that should facilitate the safe integration of AI-based decision-support tools into routine clinical practice.

## 1. Introduction

Acute pancreatitis (AP) is an inflammatory process caused by premature activation of proenzymes in the pancreatic acinar cells, resulting in damage to the organ parenchyma and the development of inflammation, both local and systemic [1]. The diagnosis of AP is made when two of the following three criteria are fulfilled: (1) typical clinical symptoms–acute, severe epigastric pain, often radiating to the back; (2) more than threefold increase in serum pancreatic enzyme activity above the upper normal limit; (3) imaging findings characteristic for AP [2]. AP is the most common gastroenterological cause of emergency hospitalization. Due to the significant morbidity and mortality, prompt diagnosis and treatment within 24 h of the first symptoms are crucial [3]. According to the Revised Atlanta Classification (RAC), there are three degrees of severity of acute pancreatitis: mild (MAP, no local and systemic complications), moderate (MSAP, transient organ failure and/or local complications) and severe (SAP, persistent organ failure > 48 h and/or death). The majority of patients have a mild form of the disease (80%), in which recovery is observed within a few days to a week. However, about 5–20% of patients develop severe AP, in which mortality rates range from 10% to 50% [4].

Assessing the course and severity of the disease at an early stage is therefore extremely important in planning the patient’s treatment. Patients with expected SAP require intensified treatment and hospitalization in intensive care units (ICUs). Clinical features that increase the risk of development of severe AP include: age over 55 years, obesity, chronic diseases, systemic inflammatory response syndrome (SIRS), abnormal laboratory tests (blood urea nitrogen (BUN) > 20 mg/dL or rising BUN), hematocrit (HCT) > 44% or HCT increased, increased serum creatinine, C-reactive protein (CRP) > 160 mg/L, increased procalcitonin and abnormalities in imaging tests (pleural effusions, lung infiltrates, extrapancreatic fluid collections) (Table 1) [5].

Imaging studies such as ultrasonography (USG), magnetic resonance (MR) and computed tomography (CT) with contrast-enhancement to determine the computed tomography severity index (CTSI) (Balthazar scale ≥ 7 points predicts a severe course of AP and a high risk of death) are also helpful in assessing the severity of acute pancreatitis [6]. However, routine CT or MR imaging in AP is not recommended and, if indicated, should be performed at least 48–72 h after the onset of symptoms.

In addition, other multi-component scales have been developed to predict the severity of acute pancreatitis. The APACHE II (Acute Physiology and Chronic Health Evaluation II) scale was developed to assess the condition of patients treated in ICU but, unfortunately, is not specific for AP [7]. On the basis of the harmless scale (HAPS), only an MAP can be diagnosed [8], and for example, a score of ≥2 points on the modified Marshall scale determines only organ failure (OF) but not disease prognosis [9]. The BISAP scale (bedside index for severity in acute pancreatitis) is a simple tool for early assessment of the severity of acute pancreatitis, but its sensitivity in predicting is assessed as moderate (50–70%). In the case of the Ranson scale, full scoring requires assessment of parameters at admission and after 48 h, which prevents early risk stratification. Although conventional prognostic scoring systems play an important role in the assessment of AP, their clinical utility is limited by several shortcomings. Most require numerous clinical and laboratory variables, some of which are unavailable at the time of admission, and their predictive performance remains imperfect. Moreover, several scores depend on serial measurements obtained over the first 24–48 h of hospitalization, delaying risk stratification and limiting their usefulness for guiding early clinical management.

Despite having laboratory and imaging test results or developed scoring systems, it is still difficult to accurately predict the severity of acute pancreatitis. Therefore, a research effort has been initiated to improve the prediction of the course of the disease of hospitalized patients. Today, artificial intelligence (AI) is increasingly being used in this indication. Using mathematical algorithms, AI can predict outcomes in detail based on available data and recognized correlations between them. As a result, AI found broad applications in radiology [10], as well as in predicting mortality among patients admitted to intensive care units [11]. The first studies related to the use of AI in predicting the course of AP have also been carried out, and their positive results encourage research on this topic [12].

In this review, we provide a comprehensive overview of the current evidence on the use of AI for the early prediction of AP severity. In addition, we critically examine the major methodological challenges associated with the development of predictive models and discuss potential strategies to improve their reliability, generalizability and future clinical implementation.

## 2. Prognostic Scoring Systems for Assessing the Severity of Acute Pancreatitis

### 2.1. Acute Physiology and Chronic Health Evaluation II

The APACHE II is a tool used in intensive care units to assess the severity of a patient’s condition and estimate the risk of death. The classification is based on the results of 12 routinely measured physiological parameters, including age, chronic diseases and mentality. The higher the score obtained on the scale, the poorer the prognosis. Studies have shown that APACHE II is an effective tool for predicting severe acute pancreatitis (AUC 0.78, 95% CI: 0.70–0.84); however, its use is time-consuming, limits early assessment of the patient’s condition, and is associated with lower specificity [13].

### 2.2. Computed Tomography Severity Index

The CTSI scale is a system for assessing the severity of acute pancreatitis based on computed tomography images. It combines the severity of pancreatitis (according to the Balthazar classification) with the presence and extent of pancreatic necrosis. Higher CTSI values are associated with a more severe clinical course and poorer outcomes (≥7 points predict a severe course of AP). An analysis of 28 studies showed that CTSI achieved optimal sensitivity in predicting severity at ≤48 h (AUC 0.84, 95% CI 0.78–0.89), with a specificity of 0.79 (0.76–0.82) [14]. Despite its clinical utility, CTSI has certain limitations, as it requires contrast-enhanced CT scanning, which may not be indicated in the early stages of the disease, and pancreatic necrosis may not be fully visible within the first 48–72 h of symptom onset.

### 2.3. Ranson Scale

The Ranson score is a widely used clinical system designed to assess the severity and predict the prognosis of acute pancreatitis. It is based on a set of 11 parameters assessed at two time points: on admission (five factors) and within the first 48 h of hospitalization (six factors). These parameters include demographic factors, laboratory test results, and indicators of the systemic inflammatory response. The Ranson criteria demonstrate high sensitivity in predicting severe acute pancreatitis (0.95; 95% CI: 0.87–0.98), with overall excellent discriminative ability (AUC 0.95; 95% CI: 0.93–0.97). It also shows good performance in predicting mortality (AUC 0.91) and organ failure (AUC 0.86), supporting its clinical utility in risk stratification. However, its relatively lower specificity indicates a tendency toward overestimation of disease severity. These results suggest that the Ranson criteria remain a useful and reliable tool for assessing disease severity and prognosis in patients with acute pancreatitis [15].

### 2.4. Bedside Index for Severity in Acute Pancreatitis

The BISAP score is a clinically practical scoring system designed for use within the first 24 h of hospital admission and is based on five readily available clinical parameters: blood urea nitrogen >25 mg/dL, cognitive impairment, the presence of systemic inflammatory response syndrome, age > 60 years, and the presence of pleural effusion. A score ≥ 3 points indicate a high risk of severe acute pancreatitis, multiorgan failure and death. As this scale is based on parameters assessed within the first 24 h of hospital admission, it does not take into account dynamic changes in the clinical course. This may reduce its specificity in some clinical situations. There are numerous studies comparing the accuracy of the BISAP and Ranson scales in predicting acute pancreatitis. The BISAP score demonstrated an overall predictive accuracy of approximately 76.2% for SAP, whereas the Ranson criteria achieved a higher accuracy of 82.2%. In terms of sensitivity, the Ranson score showed superior performance, identifying SAP more reliably than BISAP (97.4% vs. 69.2%). However, both scoring systems exhibited comparable specificity, with similar ability to correctly exclude severe disease (78.4% vs. 77.8%) [16].

### 2.5. Harmless Acute Pancreatitis Score

HAPS is a tool designed to identify patients with acute pancreatitis who are likely to have a mild disease course. The score uses three simple parameters: creatinine concentration, hematocrit and the presence of peritonitis on physical examination. Researchers have demonstrated that when the HAPS score is applied to identify mild acute pancreatitis, the pooled positive predictive value is approximately 83% (95% CI: 74–91%). In contrast, when used to predict the absence of mortality, HAPS demonstrated a very high positive predictive value of 98% (95% CI: 97–100%) [17]. The authors emphasize that HAPS is a clinically useful scale due to its accuracy in predicting a mild course of AP and the simplicity of the analysis.

### 2.6. Comparison of Prognostic Scales

Prognostic scoring systems differ considerably with respect to the number of required variables, ease of use, timing of assessment, and diagnostic accuracy, resulting in important trade-offs between simplicity and predictive performance (Table 2).

## 3. What Is Artificial Intelligence?

Artificial intelligence is a branch of computer science focused on the development of systems capable of performing tasks that typically require human intelligence, including reasoning and decision-making. In contemporary applications, AI predominantly relies on machine learning and deep learning techniques, which facilitate the analysis of large datasets and derivation of conclusions. During the training process, algorithms are exposed to large datasets containing input variables (e.g., clinical features, imaging data) and corresponding outcomes. Based on this data, the model learns patterns and correlation, gradually adjusting its internal parameters to improve the accuracy of its predictions. Once trained, the model can use this knowledge to make predictions for new, unknown cases. An AI system consists of several main components: data, a model, a training process and a prediction stage. In many cases, a model is built as an artificial neural network, which consists of layers of interconnected units that process information step by step. This structure allows the system to handle complex data and generate useful predictions.

## 4. Artificial Intelligence in Medicine

Artificial intelligence is becoming an increasingly important area of research in modern medicine due to its ability to improve clinical practice and patient outcomes. AI has the potential to support medical diagnostics, predict disease severity and prognosis, and assist clinicians in the decision-making process. Due to ability to identify patterns, AI-based models are being extensively studied as supportive tools across many medical specialties.

Recent studies have demonstrated the potential of artificial intelligence in cancer diagnostics through the analysis of cellular mechanical properties. Using AI, researchers were able to differentiate malignant breast cancer cells from normal breast cells with an accuracy of 93%. These findings highlight the potential of AI-assisted phonon analysis as a novel approach for minimally invasive cancer detection [18]. Another systematic review and meta-analysis evaluating over 920,000 ophthalmic images proved excellent diagnostic performance of artificial intelligence in detecting *Retinitis Pigmentosa*. AI-based models achieved sensitivity and specificity of 98.5% and 99.3%, respectively, with an AUROC of 0.999, highlighting the strong potential of AI-assisted image analysis in ophthalmologic diagnostics [19]. Artificial intelligence has also shown promise in endoscopic diagnostics. A deep neural network developed for the automatic assessment of endoscopic grading of gastric intestinal metaplasia (EGGIM) classification achieved an image accuracy of 87% and correctly identified patients requiring follow-up with 88% accuracy, with no false-negative results. These findings suggest that AI-assisted analysis of endoscopic images can support gastric cancer risk stratification and improve clinical decision-making in gastroenterology [20].

## 5. Effectiveness of AI in Predicting Severity of Acute Pancreatitis

The researchers highlighted several limitations of the scoring systems currently used to predict the severity of AP. The APACHE-II scale, for example, requires as many as 16 parameters to predict the severity of AP, which is sometimes too time-consuming to perform in normal clinical practice. Ranson’s criteria are useful after all medical data have been collected (i.e., 48 h after hospitalization). This may significantly delay both diagnosis and the initiation of appropriate treatment [21]. In addition, the outcome of CTSI is very dependent on the experience of the radiologists, and unfortunately, we cannot report repeatability in obtaining a high-quality CT description [22].

In recent years, numerous studies have attempted to compare machine learning (ML)-based models with various scales, such as the APACHE II, the Ranson criteria, HAPS, the CTSI scale or BISAP (Table 3). The studies showed that ML-based models showed better efficiency in terms of AUROC, sensitivity and specificity than single scores in predicting AP severity [23].

To provide a structured overview of the current evidence, a comprehensive search of the literature was conducted to identify studies evaluating the use of artificial intelligence (AI) and machine learning (ML) for predicting outcomes in patients with AP. The search was performed in the PubMed, Scopus and Web of Science databases and covered articles published between January 2006 and April 2026. The search strategy combined terms related to AP with keywords describing AI and predictive modeling. The following search terms and their combinations were used: “acute pancreatitis”, “severe acute pancreatitis”, “machine learning”, “artificial intelligence”, “deep learning”, “neural network”, “artificial neural network”, “random forest”, “decision tree”, “XGBoost”, “explainable artificial intelligence”, “prediction”, “prognosis”, “severity”, “organ failure”, “respiratory failure”, “mortality”, and “intensive care unit”. Boolean operators (“AND”, “OR”) were applied as appropriate.

Studies were eligible for inclusion if they: (1) involved adult patients with acute pancreatitis; (2) evaluated AI, ML or deep learning-based predictive models; (3) predicted clinically relevant outcomes, including disease severity, persistent organ failure, respiratory failure, intensive care unit admission or mortality; (4) were published as full-text articles in peer-reviewed journals in English. Review articles, editorials, conference abstracts, case reports, animal studies, studies unrelated to acute pancreatitis and studies lacking predictive AI/ML models were excluded.

After removal of duplicate records, titles and abstracts were screened for relevance. Potentially eligible articles underwent full-text assessment. The final selection included studies investigating the application of machine learning algorithms for the prediction of severity, organ failure, respiratory complications, intensive care unit admission and mortality in patients with AP.

Following the overview of conventional prognostic tools and the growing role of artificial intelligence in medicine, we identified 13 studies evaluating the use of machine learning models in patients with acute pancreatitis. The characteristics and main results of these studies are summarized below. The included studies demonstrated considerable heterogeneity in terms of study design, patient populations, sample size, predicted outcomes, ML techniques and validation methods. Both retrospective and prospective investigations were included, encompassing models developed for the prediction of disease severity, organ failure, respiratory failure, ICU admission and mortality. In addition, the studies differed substantially with regard to the type and number of predictive variables, as well as the extent of internal and external validation. Such heterogeneity reflects both the complexity of acute pancreatitis and the rapidly expanding application of machine learning techniques in clinical research.

A study by Mofidi et al. demonstrated that an artificial neural network (ANN) significantly outperformed the APACHE II and Glasgow score (GS) in predicting progression to SAP (*p* < 0.05 and *p* < 0.01, respectively), predicting development of multiorgan dysfunction syndrome (*p* < 0.05 and *p* < 0.01) and predicting death from AP (*p* < 0.05). In addition, the accuracy (0.975), sensitivity (0.88) and specificity (0.98) of this ANN model were significantly better than GS scores (0.807, 0.45 and 0.83, respectively) and APACHE II scores (0.824, 0.67 and 0.83, respectively). The study was based on a retrospective analysis of a prospectively collected Lothian Surgical Audit database and included 664 consecutive patients with acute pancreatitis admitted between 2000 and 2004, of whom 181 (27.3%) developed severe disease and 42 (6.3%) died during the hospitalization. Although the use of consecutive patients and prospectively collected data reduced the risk of selection and information bias, the study was conducted at a single center, which may limit the generalizability of the findings. Internal validation was performed by randomly dividing the dataset into training (60%), validation (15%), and independent testing (25%) subsets, and model performance was assessed using a hold-out test set. However, no external validation was undertaken, calibration was not evaluated or reported, and no formal assessment of overfitting was performed, although the authors suggested that the relatively large sample size and limited number of input variables reduced this risk. Furthermore, insufficient reporting of the ANN architecture and model parameters limits reproducibility. Receiver operating characteristic analysis was reported for the conventional scoring systems, with AUROC values of 0.81 for APACHE II and 0.68 for the GS score in predicting severe acute pancreatitis, whereas the exact AUROC of the ANN was not reported despite its significantly superior discriminatory performance [24].

Similarly, Jin et al. analyzed disease severity using multilayer perceptron artificial neural networks (MPL-ANNs) and partial least squares discriminant analysis (PLS-DA). The study retrospectively evaluated 300 patients with acute pancreatitis admitted to the Affiliated Hospital of Southwest Medical University between 2017 and 2019, including 133 patients with mild acute pancreatitis (MAP) and 167 patients with severe acute pancreatitis (SAP, including 88 patients with moderately severe disease). Additionally, 69 healthy controls were included for comparison. Only the first episode of AP was analyzed, while patients with active malignancy, drug abuse or severe cardiovascular, respiratory, hepatic, renal or psychiatric disorders were excluded. The study was conducted at a single tertiary care center, which ensured a relatively homogeneous patient population but may limit the external validity and generalizability of the findings. Although the inclusion of consecutive patients from routine clinical practice reduced selection bias, the retrospective design introduced a risk of information bias and missing data. Indeed, the authors reported missing values that were imputed using randomly generated values derived from the estimated model, which may have introduced additional bias. The primary predicted outcome was disease severity, specifically discrimination between MAP and SAP according to the Revised Atlanta Classification. The MPL-ANN model was developed using six routinely available laboratory parameters: percentage of neutrophils (NEU-R), neutrophil count (NEU), percentage of lymphocytes (LYM-R), C-reactive protein (CRP), amylase (AMY) and pancreatic amylase (PAMY). The MPL-ANN model achieved an AUROC of 0.984 (95% CI 0.960–1.000), sensitivity of 92.7%, specificity of 93.3%, and accuracy of 93.0%, significantly outperforming the PLS-DA model (AUROC 0.912, 95% CI 0.853–0.971; *p* = 0.022). Internal validation was performed using a random split-sample approach with 70% of patients assigned to the training cohort and 30% to an independent testing cohort. However, no external validation cohort was used, calibration metrics were not reported, and no formal assessment of model overfitting was performed. Although testing set performance remained high, reliance on a single-center retrospective dataset limits reproducibility and raises concerns regarding potential optimism in model performance estimates. The authors acknowledged these limitations and recommended future multicenter prospective validation studies to confirm the robustness and clinical applicability of the proposed model [25].

Further evidence supporting the superiority of ANN-based approaches was provided by Andersson et al., who compared ANN with logistic regression (LR) and linear discriminant analysis (LDA). ANNs selected six potential risk variables as relevant for severity prediction, including duration of pain until arrival at the emergency department, serum creatinine (Cr), alanine aminotransferase (ALT), hemoglobin, white blood cell count (WBC) and heart rate (HR). The study showed that the AUROC of the ANN model was 0.92, which was significantly higher than the LR (AUROC 0.84) and APACHE II (AUROC 0.63). The model was developed using a cohort of 139 adult patients with AP treated at Lund University Hospital between 2002 and 2005, including 20 patients who developed SAP according to the Atlanta classification. The primary predicted outcome was progression to SAP. Patient data were prospectively collected, although retrospective review of medical records was performed to complete missing information. The study was conducted at a single tertiary referral center, which ensured standardized data collection but may have limited the generalizability of the findings to other healthcare settings. The recruitment strategy reduced the risk of selection bias because all eligible hospitalized patients with AP were included. However, the relatively small number of severe cases may have affected the stability of predictor selection and model estimates. Internal validation was performed using extensive five-fold cross-validation combined with bootstrap resampling and evaluation of approximately 1.4 million ANN architectures. To reduce overfitting, the authors applied weight decay regularization, ensemble learning with 100 neural networks and repeated validation procedures. Model calibration was formally assessed using the Hosmer–Lemeshow goodness-of-fit test and demonstrated excellent agreement between predicted and observed outcomes. In addition, temporal external validation was conducted using an independent cohort of 61 patients treated at the same institution between 2007 and 2009, yielding an AUROC of 0.84. Although this represents a stronger validation strategy than simple split-sample testing, the external validation remained geographically limited because it was performed in the same center. Overall, the study demonstrated good methodological quality, with a relatively low risk of overfitting and robust internal validation. However, the lack of true multicenter external validation may limit reproducibility and broader clinical applicability of the model [26].

Besides neural network-based approaches, traditional ML methods have also been investigated. In 2006, Pearce CB et al. [27] developed a logistic regression (LR)-based ML model. For the first time, they created a model that combined the admission values of selected APACHE II and CRP components to predict SAP. The study retrospectively analyzed 370 patients admitted with AP to a single United Kingdom center over a 5-year period. After excluding 105 patients because of missing data, 265 patients were included in the final analysis, of whom 68 (25.6%) developed SAP and eight (3.0%) died. The primary predicted outcome was disease severity, defined according to the Santorini Consensus and Atlanta Criteria as the presence of organ failure and/or local complications. The final model incorporated eight variables: age, CRP, respiratory rate, arterial oxygen tension on room air (pO_2_), arterial pH, serum creatinine, WBC and Glasgow Coma Scale. The model achieved an AUROC of 0.82 (SD 0.01), significantly outperforming admission APACHE II scores (AUROC 0.74; *p* = 0.0036), with a sensitivity of 87% and specificity of 71% for predicting SAP. The use of routinely available admission variables enhanced the clinical applicability of the model. However, the retrospective nature of the study introduced the potential for information bias. In addition, nearly 30% of the original cohort was excluded because of missing data, which may have increased the risk of selection bias, although the proportions of severe cases were similar in the included and excluded populations. Internal validation was performed using bootstrap resampling with optimism correction, reducing the likelihood of overestimating model performance and partially mitigating overfitting. Feature selection was conducted through repeated bootstrap-based backward elimination, resulting in a parsimonious model with eight predictors. Nevertheless, no independent external validation cohort was used, and model calibration was not formally assessed or reported. The study was conducted at a single center, which limits the generalizability and reproducibility of the findings across different healthcare settings and patient populations. Despite these limitations, the study represented one of the earliest applications of ML in AP and demonstrated that combining conventional clinical variables with ML techniques could improve early severity prediction compared with established prognostic scoring systems [27].

Subsequent studies focused on identifying clinically applicable predictors of SAP. In a study by Hong et al., important predictors of SAP were recognized using logistic regression analysis. The study was based on a retrospective single-center cohort of 420 patients with AP admitted within 72 h of symptom onset to the First Affiliated Hospital of Wenzhou Medical College between 2007 and 2009. Patients with chronic pancreatitis, pancreatic malignancy or ERCP-related pancreatitis, significant comorbidities affecting pleural effusion or renal function, transferred patients and cases with incomplete data were excluded. Although these strict eligibility criteria improved data quality and reduced potential confounding, they may have introduced selection bias and limited the representativeness of the study population. Patients were randomly assigned to training (n = 280) and testing (n = 140) cohorts, providing a robust internal validation strategy. The primary predicted outcome was the development of SAP. Multivariable logistic regression identified SIRS, pleural effusion, serum calcium, and blood urea nitrogen (BUN) as independent predictors of SAP, while the final CART model incorporated pleural effusion, serum calcium and BUN. The model demonstrated excellent discriminatory performance, achieving an AUROC of 0.84 (95% CI 0.79–0.90) in the training cohort and 0.86 (95% CI 0.79–0.94) in the independent test cohort, outperforming APACHE II (AUROC 0.68 and 0.69, respectively; *p* < 0.001 and *p* = 0.005). Internal validation was performed using a randomly selected test dataset and the model maintained identical diagnostic accuracy (89.3%) in both cohorts, suggesting good reproducibility. Furthermore, the authors reported that predicted outcomes were reproduced consistently in the validation cohort, indicating satisfactory calibration. However, no external validation cohort from another institution was used, limiting assessment of model transportability and generalizability. The retrospective design and reliance on CT-detected pleural effusions may also introduce information and selection biases. Notably, the AUROC in the validation cohort slightly exceeded that observed in the training cohort, which the authors acknowledged could reflect either strong model performance or substantial similarity between datasets [28].

Yang et al. constructed a predictive model using the decision tree method, and this model was applied to the test group to assess its validity. The study was based on a retrospective analysis of 1308 consecutive patients with AP treated at a single tertiary referral center between 2008 and 2013. After applying strict eligibility criteria, including admission within 36 h of symptom onset, age > 18 years, and the absence of previous pancreatitis and pre-existing cardiac, respiratory or renal failure, 603 patients were included in the final analysis. Among them, 103 patients (17.2%) developed SAP, while the overall 30-day mortality was 2.5%. The primary predicted outcome was the development of SAP. The use of a large, consecutively recruited cohort reduced the risk of selection bias. However, exclusion of more than half of the initially screened patients may have limited the representativeness of the study population and introduced spectrum bias. Furthermore, the retrospective single-center design increases the risk of information bias and limits generalizability to other healthcare settings. Internal validation was performed by randomly dividing the cohort into a training set (n = 402) and an independent test set (n = 201) with comparable baseline characteristics between groups. The final CART model incorporated three routinely available variables obtained within 12 h of admission: serum creatinine, lactate dehydrogenase (LDH), and oxygenation index. In the training cohort, the model achieved a sensitivity of 80.9%, specificity of 90.0%, and overall accuracy of 88.5%, while in the independent test cohort, sensitivity and specificity increased to 88.6% and 90.4%, respectively. Receiver operating characteristic analysis demonstrated an AUROC of 0.855 (95% CI 0.814–0.896), which was numerically higher than that of the BISAP score (AUROC 0.802, 95% CI 0.752–0.853), although the difference did not reach statistical significance (*p* = 0.098). Internal validation suggested good reproducibility, as model performance remained stable in the independent test cohort. However, no formal calibration assessment was reported, and neither calibration plots nor goodness-of-fit statistics were provided. Importantly, no external validation cohort was used, and the authors acknowledged that prospective multicenter studies are required to confirm the robustness and transportability of the model. Consequently, despite promising discrimination and simplicity, the study remains at moderate risk of bias due to its retrospective design, single-center setting, lack of external validation and absence of formal calibration analysis [29].

Beyond severity prediction alone, AI models have also been applied to forecast specific complications of AP. Hong et al. developed an artificial neural network to predict persistent organ failure in patients with AP. The study retrospectively included 312 patients admitted within 72 h of symptom onset to a single tertiary university hospital. Persistent organ failure was defined as cardiovascular, respiratory or renal failure lasting more than 48 h and represented the main predicted outcome. Among the included patients, 48 developed persistent organ failure, and respiratory failure was the most frequent form. The final ANN model used five variables: age, hematocrit, serum glucose, BUN and serum calcium. It achieved an AUROC of 0.96 ± 0.02, which was significantly higher than that of the logistic regression model (AUROC 0.88 ± 0.03) and APACHE II score (AUROC 0.83 ± 0.03; both *p* < 0.001). The model also showed high diagnostic accuracy (96.2%), sensitivity (81.3%), and specificity (98.9%). Clinically, the ANN increased the post-test probability of persistent organ failure to 93% when positive and reduced it to 3% when negative. Internal validation was performed using five-fold cross-validation, and overfitting was addressed by applying an overfit penalty and selecting variables through univariate and sensitivity analyses. However, no independent external validation cohort was used, and calibration was not formally assessed with calibration plots or goodness-of-fit statistics. The study was single-center and retrospective, which increases the risk of selection and information bias and limits generalizability to community hospitals or other healthcare systems [30].

With the increasing availability of large clinical databases, ML models have also been trained on ICU-derived datasets. Ding et al. first used the Medical Information Mart for Intensive Cart III (MIMIC-III) database to predict AP hospital mortality. The database contained ICU admissions from Beth Israel Deaconess Medical Center between 2001 and 2012. After excluding patients younger than 18 years and those with more than 5% missing data, 337 patients with AP were included, of whom 38 (11.2%) died during hospitalization. The primary predicted outcome was mortality. The use of a large ICU database reduced the risk of referral bias and increased the heterogeneity of the study population. However, inclusion was limited to critically ill patients admitted to the ICU, which may restrict the applicability of the findings to the broader population of patients with AP. Furthermore, the retrospective nature of the study exposed it to information bias and potential residual confounding resulting from variables unavailable in the database. Twelve clinical and laboratory variables collected within the first 24 h after admission were used to develop the ANN model, including age, ALT, total bilirubin, MB isoenzyme (CK-MB), prothrombin time (PT), WBC, amylase, total calcium, creatinine, hematocrit, lactate, and lipase. Internal validation was performed using a random split-sample approach, with 80% of patients allocated to the training cohort and 20% to the validation cohort. Baseline characteristics were comparable between the two groups, suggesting adequate internal validation. The ANN achieved an AUROC of 0.769, outperforming logistic regression (AUROC 0.607), the Ranson score (AUROC 0.652) and the SOFA score (AUROC 0.401). Multivariable logistic regression identified ALT, WBC and total calcium as independent predictors of mortality, whereas ANN variable importance analysis highlighted total bilirubin, amylase, ALT and creatinine as the most influential features. Despite its superior discrimination, no external validation cohort was used, calibration was not assessed or reported and no formal calibration metrics or plots were provided. The authors did not perform a dedicated overfitting analysis. However, the use of a separate validation cohort partially reduced this risk. Reproducibility was enhanced by the use of a publicly accessible database and detailed reporting of input variables, although transportability to non-ICU populations remains uncertain. The authors acknowledged that the relatively small sample size, retrospective design, predominance of American patients and lack of multicenter external validation limited the generalizability of the model [31].

More recently, large multicenter machine learning projects have further expanded the applicability of AI in AP. The EASY-APP study represents one of the largest machine learning projects in AP to date. Unlike many previous AI studies based on retrospective single-center datasets, this was a multinational, multicenter, prospective observational study involving 28 centers across 15 countries. The derivation cohort included 1184 patients enrolled within 12 h of hospital admission, while external validation was performed in an independent cohort of 3164 patients from four international pancreatic centers, resulting in a total study population approaching 5000 patients. The primary outcome was the development of SAP. The prospective design, predefined protocol, international recruitment strategy and four-stage data quality control process substantially reduced the risk of selection and information bias. Furthermore, the inclusion of consecutive patients from multiple healthcare systems enhanced the representativeness and generalizability of the findings. Nevertheless, the study population was enriched with patients assessed within 12 h of admission and excluded cases with insufficient data quality, which may have introduced a degree of selection bias. Internal validation was performed using four-fold cross-validation, while external validation was conducted in four independent international cohorts, representing a major methodological strength compared with earlier AP prediction studies. To address model instability and estimate prediction uncertainty, the authors additionally applied bootstrap resampling with 100 iterations and generated confidence intervals for both model performance and individual predictions. The best-performing model was an XGBoost classifier, achieving a cross-validated AUROC of 0.81 (95% CI 0.776–0.842) and an accuracy of 89.1%. External validation demonstrated consistent discrimination, with AUROC values ranging from 0.72 to 0.79 across independent cohorts. The most influential predictors identified by SHAP analysis included respiratory rate, body temperature, abdominal guarding, sex, age, glucose concentration, creatinine and blood urea nitrogen. Importantly, the use of cross-validation, external validation, oversampling techniques (SMOTE) and explainable AI methods reduced the risk of overfitting and improved model robustness and reproducibility. However, formal calibration analysis was not reported, and therefore, agreement between predicted and observed risk could not be fully assessed. A further limitation was the binary classification strategy, which grouped mild and moderately severe AP together and may have reduced discriminatory performance for intermediate-risk patients. Overall, the EASY-APP study provides one of the strongest methodological frameworks among AI-based AP prediction models currently available and demonstrates good transportability across different international populations [32].

Further progress in this field was demonstrated by İnce et al., who conducted an interesting study in which they used ML to predict course severity, survival and ICU requirements. The study was a retrospective single-center analysis conducted at Bezmialem Vakif University in Turkey and included 1334 adult patients with AP treated between 2010 and 2020. In total, 216 patients were excluded because of incomplete data, while patients with chronic pancreatitis, previous pancreatic surgery, pancreatic cancer, or contraindications to contrast-enhanced imaging were also excluded. Severity was classified according to the Revised Atlanta Classification, with 57.1% of patients presenting with mild AP, 20.4% with moderately severe AP, and 22.5% with severe AP. ICU admission occurred in 4.6% of patients, and overall mortality was 9.9%. The study aimed to predict three clinically relevant outcomes: disease severity, ICU requirement and survival. Although the large cohort and standardized diagnostic criteria strengthened the study, the retrospective design and exclusion of patients with missing data introduced a risk of selection and information bias. Furthermore, recruitment from a single tertiary referral center may limit the generalizability of the findings to other healthcare systems and populations. The authors developed a Gradient Boost Tree Model using 13 demographic, clinical, etiologic and laboratory variables available at admission and evaluated performance both with and without incorporation of the 48 h Balthazar-CTSI score. Internal validation was performed using a structured approach consisting of a 90% ML dataset and an independent 10% validation cohort, combined with stratified sampling, 70/30 train–test splitting, five-fold cross-validation, and SMOTE oversampling to address class imbalance. No external validation cohort was used. The model demonstrated good discriminatory performance for all three outcomes. For prediction of SAP, the AUROC was 0.896 without CTSI and 0.914 with CTSI. For ICU admission, AUROC values were 0.859 without CTSI and 0.885 with CTSI. For survival prediction, AUROC values reached 0.978 without CTSI and 0.910 with CTSI. Variable importance analysis identified creatinine, etiology, age, HTC, BMI and BUN as the most influential predictors of disease severity when CTSI was not included, whereas Balthazar-CTSI became the dominant predictor when incorporated into the model. The use of cross-validation, an independent validation dataset and SMOTE likely reduced the risk of overfitting and improved model robustness. However, formal calibration analyses, calibration plots, Brier scores or goodness-of-fit statistics were not reported, limiting assessment of agreement between predicted and observed outcomes. Reproducibility was enhanced by detailed reporting of predictors and ML procedures, although the lack of external validation remains a major limitation. Overall, the study demonstrated promising predictive performance but remains at moderate risk of bias owing to its retrospective design, single-center setting and absence of independent multicenter validation [33].

A recent prospective cohort study developed and validated an explainable ML model for mortality prediction in patients with infected pancreatic necrosis (IPN). The study by Ning et al. represents one of the most methodologically rigorous applications of ML. Using a prospective derivation cohort of 344 consecutive patients with IPN and an independent external validation cohort of 132 patients from another tertiary center, the authors developed and validated an explainable random survival forest model for predicting 90-day mortality. The model demonstrated excellent discrimination, achieving a concordance index (C-index) of 0.863 (95% CI 0.854–0.875) in the derivation cohort and 0.857 (95% CI 0.850–0.865) in the external validation cohort, indicating strong generalizability. Because the model was developed for time-to-event prediction, discrimination was assessed using the concordance index and time-dependent AUC rather than conventional AUROC. Internal validation was performed using 1000-bootstrap resampling, while calibration was formally assessed using calibration curves and integrated Brier scores, demonstrating good agreement between predicted and observed outcomes. The use of SHAP analysis further enhanced model interpretability by identifying multiple organ failure, APACHE II score ≥ 20, prolonged organ failure, bloodstream infection, and hemorrhage as major predictors of mortality. Despite these strengths, the study was conducted exclusively in Chinese tertiary referral centers, which may limit the generalizability of the findings to other healthcare settings and patient populations [34].

Similarly, in a multicenter study involving 1802 patients, several ML algorithms were evaluated, with the CatBoost model demonstrating the best predictive performance. The final model achieved an AUC of 0.835 in the test cohort and 0.782 in external validation. The study aimed to predict in-hospital mortality among patients with AP admitted to the ICU, making mortality the primary outcome. The development cohort consisted of 1611 patients derived from the MIMIC-IV and MIMIC-III databases, while external validation was performed in an independent cohort of 199 patients from Beijing Chaoyang Hospital. The study was retrospective in nature, relying primarily on large critical care databases, although the inclusion of an external cohort strengthened the assessment of model generalizability. Internal validation was performed using an 80/20 split of the MIMIC-IV dataset, followed by testing in an independent MIMIC-III cohort, and model training incorporated k-fold cross-validation to reduce overfitting. Furthermore, external validation in a geographically distinct cohort provided additional evidence of robustness. Calibration analysis demonstrated good agreement between predicted and observed mortality, with the optimized CatBoost model outperforming logistic regression, random forest, and XGBoost models. The use of multiple independent datasets, feature selection with LASSO, hyperparameter optimization, and external validation reduced the risk of overfitting and improved reproducibility. Nevertheless, the study remains subject to several potential sources of systematic bias. Patient selection was restricted to critically ill ICU populations, limiting applicability to the broader spectrum of acute pancreatitis. Additionally, the retrospective design, exclusion of patients with substantial missing data and reliance on routinely collected electronic health records may have introduced selection bias, information bias, and residual confounding. Despite these limitations, the multicenter design, large sample size and external validation support the reliability and clinical relevance of the proposed model [35].

Machine learning has also shown potential in predicting systemic complications associated with AP. In a study by Zhou et al., several ML algorithms were developed to predict acute respiratory failure (ARF). AUC values were 0.86 and 0.87 in the training and validation cohorts. The model used six clinical variables, including calcium, albumin, white blood cell count, anion gap, and blood urea nitrogen. The primary outcome was the development of ARF during hospitalization in patients with AP. This retrospective study included 4527 patients identified from the MIMIC-IV critical care database, of whom 445 (9.8%) developed ARF. Patients were randomly divided into training (70%) and validation (30%) cohorts. Feature selection was performed using both random forest and LASSO regression with 10-fold cross-validation, resulting in six final predictors (calcium, albumin, glucose-to-lymphocyte ratio, white blood cell count, anion gap and blood urea nitrogen). Several ML models were evaluated, including logistic regression, decision tree, k-nearest neighbors, naïve Bayes, and XGBoost, with XGBoost demonstrating the best discriminative performance (AUROC 0.862 in the training cohort and 0.869 in the validation cohort). Internal validation was strengthened through random data splitting, 10-fold cross-validation during feature selection, assessment of multicollinearity and the use of synthetic minority oversampling to address class imbalance and reduce overfitting. Calibration curves were also presented and showed superior agreement between predicted and observed outcomes for the XGBoost model compared with alternative algorithms. However, despite the large sample size, the study lacked true external validation, as both development and validation cohorts originated from the same database. Furthermore, the retrospective design, exclusion of patients with substantial missing data and restriction to critically ill ICU patients may have introduced selection bias and limited generalizability to the broader population of patients with acute pancreatitis. Nevertheless, the large cohort size, rigorous feature selection strategy and consistent model performance across training and validation datasets support the robustness of the proposed prediction model [36].

## 6. Limitations and Future Perspectives of Artificial Intelligence in Acute Pancreatitis

Although the research results are promising, they require further improvement before widespread clinical implementation. Not all studies were conducted on sufficiently large patient cohorts, and many relied on retrospective, single-center datasets, which increase the risk of selection and information bias and limit the generalizability of the findings. Researchers should pay greater attention to data management in future studies by providing detailed descriptions of data sources, patient selection, preprocessing procedures, handling of missing data, feature engineering and model evaluation to improve transparency and reproducibility [37]. Although most investigators performed internal validation and feature selection to reduce the risk of overfitting, independent external validation was absent in many studies. Future research should therefore focus on validating ML models in geographically and ethnically diverse populations using prospective multicenter cohorts [38].

Another important limitation is the considerable heterogeneity among published studies. Different investigators used diverse patient populations, inclusion criteria, predictor variables, machine learning algorithms, outcome definitions and performance metrics, making direct comparisons between models difficult. Moreover, only a minority of studies formally assessed model calibration despite calibration being essential for evaluating the agreement between predicted and observed risk before clinical implementation [39]. Future studies should follow standardized reporting guidelines, such as TRIPOD-AI and PROBAST-AI, to improve methodological quality and facilitate comparison across studies [38,40]. In addition, subsequent prospective studies evaluating the clinical impact of AI-based decision-support systems should adhere to the CONSORT-AI and SPIRIT-AI guidelines, which were specifically developed to improve the design, conduct and reporting of clinical trials involving AI interventions [37,41]. Wider adoption of these frameworks may enhance transparency, reproducibility and the quality of evidence supporting AI implementation in clinical practice.

Another increasingly important aspect of AI research is model interpretability [42]. Although many machine learning algorithms demonstrate excellent predictive performance, the complexity of their decision-making processes may limit transparency and reduce clinician confidence in their predictions. Consequently, explainable AI has emerged as a promising approach to improve model transparency and facilitate the clinical adoption of AI-based decision-support systems [43]. Recent studies have increasingly incorporated explainability techniques such as SHapley Additive exPlanations to identify the variables contributing most strongly to model predictions and to provide clinically meaningful insights into model behavior [44,45]. Future research should continue to integrate explainability methods alongside traditional performance metrics to ensure that AI models are not only accurate but also transparent, interpretable, and trustworthy for clinical decision-making [46].

Lack of external validation was not the only factor limiting the reliability of the published models. Only a few authors made their predictive models, datasets or source code publicly available. This considerably limits independent validation, reproducibility and further development of these algorithms. Future researchers should promote open science by sharing models, source code and anonymized datasets to facilitate collaborative research and accelerate clinical translation [47].

Finally, despite excellent discrimination reported by many models, few studies have evaluated whether AI-assisted prediction improves real-world clinical decision-making or patient outcomes. Most models remain at the stage of technical validation, and prospective impact studies assessing their influence on treatment strategies, resource allocation and clinical outcomes are still lacking [48].

Future research should focus on prospective clinical implementation studies to determine whether AI-assisted risk stratification can improve patient outcomes, optimize resource utilization and support personalized treatment strategies in routine practice [49]. In addition, regulatory, legal and ethical aspects of AI implementation require further attention [50]. Issues related to data privacy, algorithm transparency, accountability, cybersecurity and regulatory approval processes must be addressed before widespread clinical adoption can be achieved.

Emerging developments in AI may further enhance the predictive performance and clinical applicability of ML models in AP. Multimodal AI systems capable of integrating heterogeneous data sources, including clinical variables, laboratory results, radiological imaging, electronic health records and omics data, may provide more comprehensive and individualized risk assessment [51]. Federated learning represents another promising approach by enabling collaborative model development across multiple institutions without requiring the transfer of sensitive patient data, thereby improving data privacy, model robustness and generalizability [52]. Furthermore, foundation models and large-scale pretrained architectures may facilitate knowledge transfer across healthcare domains and improve predictive performance, particularly in settings with limited disease-specific datasets [53].

In conclusion, ML has shown great potential as a clinical decision-support tool. AI demonstrated high accuracy in predicting disease severity, complications, intensive care unit admission, organ failure and mortality in patients with acute pancreatitis. However, current evidence is limited by methodological heterogeneity, insufficient external validation, limited assessment of calibration and the lack of prospective implementation studies.

Future research should prioritize standardized methodology, transparent reporting, explainable AI approaches, multicenter external validation, open science practices and prospective clinical trials to develop robust and trustworthy ML models that can be safely integrated into routine clinical practice.

## 7. Conclusions

Prognostic scoring systems play an important role in assessing the severity of AP by facilitating the early identification of patients at increased risk of complications, organ failure and death. Despite their widespread use, these tools have several important limitations. Their predictive performance remains moderate, many require prolonged clinical observation or the collection of numerous variables, and some rely on imaging studies that are not routinely available during the early phase of the disease. For example, although the computed tomography severity index provides valuable information on pancreatic necrosis and local complications, its clinical utility is limited in the initial stage of AP because contrast-enhanced computed tomography is usually not performed immediately after admission.

These limitations have stimulated growing interest in AI and ML as novel approaches to early risk prediction. The studies included in this review demonstrate that ML models can accurately predict clinically relevant outcomes, including disease severity, organ failure, respiratory failure, intensive care unit admission and mortality, while simultaneously integrating a large number of demographic, clinical, laboratory and imaging variables. In several studies, AI models outperformed conventional prognostic scores, highlighting their potential as clinical decision-support tools.

However, the current evidence also indicates that most AI models remain at the stage of technical validation. Methodological heterogeneity, the predominance of retrospective single-center studies, limited external validation, insufficient calibration assessment and the lack of prospective implementation studies currently restrict their routine clinical application. Future research should therefore prioritize multicenter prospective validation, standardized reporting, transparent model development and the integration of explainable AI methods to improve interpretability and clinician confidence. Emerging approaches, such as multimodal AI integrating clinical, laboratory, imaging and omics data, may further improve prediction accuracy and enable more comprehensive risk stratification. In addition, federated learning could facilitate the development of robust multicenter models while preserving patient privacy by allowing institutions to collaborate without sharing raw data. The rapid development of foundation models may also enhance predictive performance through transfer learning and adaptation to diverse clinical settings. Nevertheless, prospective implementation studies are still needed to determine whether AI-assisted prediction improves clinical decision-making and patient outcomes in routine practice. Furthermore, successful clinical implementation will require compliance with evolving regulatory frameworks, continuous model monitoring and careful evaluation of algorithm transparency, safety, fairness and potential bias. Addressing these challenges will be essential for translating AI-based prediction models into reliable tools that complement, rather than replace, established prognostic scoring systems and support personalized management of patients with acute pancreatitis.

## Figures and Tables

**Table 1:** Clinical features that increase the risk of development of severe AP.

Category	Risk Factors Associated with Severe Acute Pancreatitis
Demographic factors	Age > 55 years
Anthropometric factors	Obesity
Comorbidities	Chronic diseases
Clinical features	Systemic inflammatory response syndrome (SIRS)
Laboratory abnormalities	BUN > 20 mg/dL or rising BUN
	HCT > 44% or increasing HCT Elevated serum creatinine CRP > 160 mg/dL Elevated procalcitonin
Imaging abnormalities	Pleural effusions Pulmonary infiltrates Extrapancreatic fluid collections

**Table 2:** Comparison of prognostic scales.

Prognostic Scale	Advantages	Limitations
APACHE II	- High predictive accuracy for severe acute pancreatitis and organ failure; - Applicable at admission and during hospitalization; - Dynamic reassessment possible.	- Time-consuming and complex; - Requires multiple physiological measurements; - Lower specificity; - Less practical in routine emergency settings.
CTSI	- Enables assessment of pancreatic necrosis and local complications; - Useful for imaging-based severity evaluation; - Good prognostic value in advanced disease.	- Requires contrast-enhanced computed tomography; - Limited utility during the early phase of disease; - Exposure to radiation and contrast agents; - Depends on the skills and experience of the radiologist.
Ranson scale	- High sensitivity for predicting severe acute pancreatitis and mortality; - Widely validated and commonly used in clinical studies.	- Requires 48 h for complete assessment, limiting early clinical decision-making; - Relatively complex; - Lower specificity.
BISAP	- Simple and easy to calculate; - Can be assessed within the first 24 h of admission; - Useful for early risk stratification; - Requires only routinely available clinical parameters.	- Lower sensitivity compared with more complex scoring systems; - Limited ability to assess local pancreatic complications.
HAPS	- Rapid and simple bedside assessment; - Useful for identifying patients with mild acute pancreatitis.	- Limited ability to predict severe disease and complications; - Lower sensitivity for severe acute pancreatitis.

**Table 3:** Detailed characteristics of the studies published in 2006–2025.

Year	Author	Number of Patients	ML Model	Validation Method	Predictive Feature	AUROC
2006	Pearce CB et al.	Training set: 200 Test set: 65	LR	Bootstrapping	Age; CRP; RR; PaO_2_; pH; Cr; WBC; GCS score	0.82
2007	Mofidi et al.	Training set: 398 Validation set: 100 Test set: 166	ANN	Random splitting	Age; hypotension unresponsive to fluid resuscitation; SIRS; SaO_2_; LDH; glucose; Ure; Ca^2+^; HCT; WBC	Not specified
2011	Hong et al.	Training set: 280 Test set: 140	CART, LR	NA	Pleural effusion; Ca^2+^; BUN	CART-0.86 LR-0.90
2011	Andersson et al.	Training set: 139 Test set: 61	ANN, LR	5-fold cross-validation	Duration of pain; Cr; Hb; ALT; RR; WBC	ANN-0.92 LR-0.84
2013	Hong et al.	Training set: 250 Test set: 62	ANN, LR	5-fold cross-validation	Age; HCT; BUN; Ca^2+^; glucose	ANN-0.96 LR-0.88
2015	Yang et al.	Training set: 402 Test set: 201	DT	NA	Cr; LDH; OI	0.855
2021	Jin X et al.	Training set: 214 Test set: 86	ANN, PLS-DA	Cross-validation	NEU-R; NEU; LYM-R; AMY; PAMY; CRP	MPL-ANN-0.98 PLS-DA-0.91
2021	Ding et al.	Training set: 269 Test set: 68	ANN, LR	NA	Age; ALT; TB; CK-MB; PT; WBC; AMY; Ca2^+^; Cr; HCT; lactate; lipase	ANN-0.77 LR-0.60
2022	Kui B. et al.	Training set: 1184 Validation set: 3164	DT, RF, LR, SVM, CatBoost, XGBoost	4-fold cross-validation	Cr, glucose, RR, BUN, WBC and gender	XGBoost-0.81 CatBoost-0.81 RF-0.8 SVM-0.79 LR-0.77 DT-0.74
2023	İnce et al.	Training set: 840 Validation: 134 Testing set: 360	GBTM	5-fold cross-validation	Cr, etiology, age, Hct, BMI, BUN and weight or if CTSI were used: CTSI, Cr, etiology, age, and Hct	0.90 without CTSI 0.91 with CTSI
2024	Zhou et al.	Training set: 3169 Validation: 1358	LR, DT, KNN, NB, XGBoost	Random splitting	Ca^2+^, albumin, glucose-to-lymphocyte ratio, WBC, anion gap, BUN	XGBoost-0.87 NB-0.86 LR-0.85 KNN-0.83 DT-0.78
2025	Ning et al.	Training set: 344 Validation: 132	RF, Cox proportion al hazards, XGBoost, SVM, DeepSurv, DeepHit, GBM	Internal-bootstrap method; External-independent test cohort	Multiple organ failure, APACHE II score ≥ 20, organ failure duration ≥ 21 days, bloodstream infection, time from disease onset to first intervention < 30 days, BISAP score ≥ 3, critical acute pancreatitis, age ≥ 50 years, hemorrhage	C-index: RF-0.86 Cox-0.85 XGBoost-0.84 GBM-0.84 DeepSurv-0.84 DeepHit-0.83 SVM-0.82
2025	Wei et al.	Training set: 650 Validation: 162 Testing set: 799	LR, KNN, DT, RF, SVM, XGBoost, AdaBoost, GBTM, MLP, LightGBM, CatBoost	k-fold cross-validation	MV, BUN, age, CRRT, sepsis, lactate, SpO_2_, INR, WBC, DBP, temperature, AST, ALT	CatBoost-0.84 RF-0.84 AdaBoost-0.83 XGBoost-0.83 LightGBM-0.83 GBDT-0.82 KNN-0.82 LR-0.81 SVM-0.79 MLP-0.79 DT-0.75

Abbreviations: alanine aminotransferase (ALT); aspartate aminotransferase (AST); amylase (AMY); artificial neural network (ANN); Acute Physiology and Chronic Health Examination II (APACHE II); bedside index of severity in acute pancreatitis (BISAP); body mass index (BMI); blood pressure (BP); blood urea nitrogen (BUN); serum calcium (Ca^2+^); classification and regression tree (CART); continuous renal replacement therapy (CRRT); creatine kinase, MB isoenzyme (CK-MB); creatinine (Cr); C-reactive protein (CRP); decision tree (DT); diastolic blood pressure (DBP); gradient boost tree model (GBTM); Glasgow Coma Scale (GCS); hemoglobin (Hb); hematocrit (HCT); heart rate (HR); international normalized ratio (INR); K-nearest neighbors (KNN); lactate dehydrogenase (LDH); low-density lipoprotein (LDL); serum lipase (LPS); logistic regression (LR); lymphocyte percentage (LY%); percentage of lymphocytes (LYM-R); multilayer perceptron (MLP); mechanical ventilation (MV); naive Bayes (NB); not available (NA); neutrophil count (NEU); percentage of neutrophils (NEU-R); pancreatic amylase (PAMY); alveolar oxygen partial pressure (PaO2); arterial Ph (pH); platelet (PLT); partial least squares discrimination (PLS-DA), prothrombin time (PT); random forest (RF); respiratory rate (RR); surface area density (SAD); arterial oxygen saturation (SaO2); systemic inflammatory response syndrome (SIRS); support vector machine (SVM); total bilirubin (TB); temperature (Temp); triglyceride (TG); thrombin time (TT); urea (Ure); white blood cell (WBC); extreme gradient boosting (XGBoost).

## Data Availability

No new data were created or analyzed in this study. Data sharing is not applicable to this article.
